# Multi-year progesterone profiles during pregnancy in baleen of humpback whales (*Megaptera novaeangliae*)

**DOI:** 10.1093/conphys/coab059

**Published:** 2021-07-28

**Authors:** Carley L Lowe, Kathleen E Hunt, Matthew C Rogers, Janet L Neilson, Jooke Robbins, Christine M Gabriele, Suzie S Teerlink, Rosemary Seton, C Loren Buck

**Affiliations:** 1 Department of Biological Sciences, Northern Arizona University, Flagstaff, AZ 86011, USA; 2 Department of Biology, George Mason University and Smithsonian-Mason School of Conservation, Front Royal, VA 22630, USA; 3 Alaska Fisheries Science Center Auke Bay Laboratories, NOAA, National Marine Fisheries Service, Juneau, AK 99801, USA; 4 Humpback Whale Monitoring Program, Glacier Bay National Park and Preserve, Gustavus, AK 99826, USA; 5 Center for Coastal Studies, Provincetown, MA 02657, USA; 6 Alaska Regional Office, NOAA Fisheries, Juneau, AK 99801, USA; 7 College of the Atlantic, Bar Harbor, ME 04609, USA

## Abstract

Understanding calving rates of wild whale populations is critically important for management and conservation. Reproduction of humpback whales (*Megaptera novaeangliae*) is difficult to monitor and, even with long-term sighting studies, basic physiological information such as pregnancy rates and calving intervals remain poorly understood in many populations. We hypothesized that pregnant whales have sustained elevations in baleen progesterone that temporally correlate with gestation. To test this hypothesis, baleen progesterone profiles from two adult female North Pacific humpbacks, both with extensive sighting records and documented pregnancies, were compared to those of a nulliparous female (adult female never seen with a calf) and a juvenile male. Baleen specimens recovered during necropsy were subsampled every 2 cm from the base to the tip of the plate, with each interval representing 30–45 days of growth. Homogenized baleen powder was assayed for progesterone using enzyme immunoassays. The date of growth of each sampling location on the baleen plate was estimated based on stable isotope analysis of annual δ^15^N cycles. Progesterone profiles from both pregnant whales showed sustained high progesterone content (>350 ng/g) in areas corresponding to known pregnancies, inferred from calf sightings and post-mortem data. The younger female, estimated to be 13 years old, had higher progesterone during pregnancy than the 44.5 year old, but levels during non-pregnancy were similar. The nulliparous female and the male had low progesterone throughout their baleen plates. Baleen hormone analysis can determine how progesterone concentrations change throughout gestation and has potential for estimating age at first reproduction, pregnancy intervals, failed pregnancies and early calf mortality. Understanding rates of calving and current and historic reproductive patterns in humpbacks is vital to continuing conservation measures in this species.

## Teaser

Baleen of two pregnant humpback whales contains areas of high progesterone that span the duration of pregnancy (determined from sighting records), while baleen from a non-pregnant female and juvenile male had low progesterone through the baleen. Thus, baleen hormone analysis can identify pregnancies and inter-calving intervals of female humpback whales.

## Introduction

The ability to assess basic reproductive parameters, such as sexual maturity and pregnancy state, is important for understanding wildlife biology and population dynamics. However, such assessments are exceedingly difficult for living baleen whales because they have few definitive external indicators of reproductive state beyond the presence of a dependent calf. In other vertebrates, observational data can be supplemented with physical examinations or hormone levels assessed via blood serum, but these data are not currently obtainable from free swimming whales. In recent years, researchers have attempted to use faeces, blubber and respiratory vapour (‘blow’) instead of blood to measure steroid hormones relevant for addressing questions of stress and reproductive physiology in baleen whales (Hunt *et al.*, 2013; Rolland *et al.*, 2005; Trumble and Usenko, 2015; Pallin *et al.*, 2018). However, most of these samples can only measure a single point in time in the whale’s life; repeated sampling of the same individual is often impossible, and retrospective analysis is rarely feasible.

Baleen comprises the keratinous overlapping plates that hang from the upper jaw, used for filter feeding (Fudge *et al.*, 2009; Hunter, 1788; Tullberg, 1883). Baleen grows continuously and slowly, thus baleen analysis yields longitudinal data spanning years, with weekly to monthly temporal resolution at any given sampling point (Aguilar and Borrell, 1991; Hunt *et al.*, 2013; Hunt *et al.*, 2014; Hunt *et al.*, 2016a; Hunt *et al.*, 2016b; Lysiak *et al.*, 2018; Schell, 2000). Stable isotopes (SIs) and steroid hormones are incorporated into the baleen as it grows, such that a single plate of baleen holds a time series spanning multiple years or even decades in some species (Hobson *et al.*, 2004; Hunt *et al.*, 2014; Hunt *et al.*, 2018; Matthews and Ferguson, 2015), making it an ideal tissue type for examination of long-term physiological trends. Baleen can unfortunately only be obtained from dead animals, and individuals cannot be targeted for sample collection so samples can be rare; however, when baleen is available, it offers the benefit of detailed longitudinal information on an individual. These multi-year analyses of individuals can be an important first step to determining how the reproductive physiology of large whales changes during life events such as pregnancy.

There is evidence that elevated progesterone in baleen could be used to diagnose pregnancy and calving intervals in baleen whales. Studies that have examined circulating blood levels of progesterone in mammals during pregnancy show a pattern of gradual stepwise increase during gestation followed by a sharp decrease after birth [e.g. cattle (*Bos taurus*), Robertson, 1972; Stabenfeldt *et al.*, 1970; white-tailed deer (*Odocoileus virginianus borealis*), Plotka *et al.*, 1977); mice (*Mus musculus*), Murr *et al.*, 1974; Asian elephants (*Elephas maximus*), Olsen *et al.*, 1994]. Captive killer whales (*Orcinus orca*, Katsumata *et al.*, 2005, Katsumata, 2010) and bottlenose dolphins (*Tursiops truncatus* and *Tursiops truncatus gili*; Bergfelt *et al.*, 2011; Cornell *et al.*, 1987), both odontocetes, show sharp increases in plasma progesterone shortly after conception and then a gradual decrease before parturition. Thus, progesterone patterns can often diagnose pregnancy, at least in species that do not have obligate pseudopregnancy. Pseudopregnancy, a sustained elevation in circulating progesterone without the presence of a foetus, is known to occur in many delphinid species (e.g. dolphins, killer whales and false killer whales; Dierauf and Gulland, 2001). These pseudopregnancy analyses used odontocete species that can be held in captivity where pregnancy can be confirmed via ultrasound. It is currently unknown if baleen whales also experience pseudopregnancy, but studies of faeces, blubber, some baleen data and limited data from necropsies suggest that high progesterone levels typically correspond to true pregnancy events (Hunt *et al.*, 2014; Hunt *et al.*, 2016a; Kellar *et al.*, 2013; Pallin *et al.*, 2018). Thus, elevations in baleen progesterone might enable diagnosis of pregnancy in humpback baleen whales.

Pregnancy profiles have been recovered from species of the family Balaenidae that have exceptionally long baleen (max length, ~300 cm) [i.e. North Atlantic right whale (NARW), *Eubalaena glacialis*, Hunt *et al.*, 2016a; bowhead whale, *Balaena mysticetus*, Kellar *et al.*, 2013]. However, to our knowledge, there have been no published reports of hormone profiles of any of the baleen whale species with shorter baleen (max length, ~65 cm) from the family Balaenopteridae (e.g. humpback whale, *Megaptera novaeangliae*; and blue whale, *Balaenoptera musculus*). It has been unclear whether the baleen growth rate (BGR) is slow enough in humpback whales to capture a full pregnancy or even multiple pregnancies (which would, in turn, enable study of inter-calving intervals; that is, number of years between pregnancies). Additionally, there may be species-specific variation in hormone patterns or baleen hormone deposition rates. In most baleen whales, the growth rate of a baleen plate can be determined by measuring the distance between peaks of the isotope nitrogen-15 (δ^15^N), which changes seasonally as the whales alternate between feeding and fasting as they migrate (Hobson and Schell, 1998; Hobson *et al.*, 2004; Lubetkin *et al.*, 2008), thus allowing for sections of baleen to be paired with corresponding sighting history events.

To physiologically validate whether progesterone in baleen can be used to diagnose pregnancy, some life history information of the individual prior to death must be known to confirm that the hormone change occurred in concert with an independently confirmed physiological state (i.e. a known pregnancy). Although there are many baleen specimens in museum collections and stranding archives, few have the corresponding biological information that is needed for establishing baseline values during various life history changes, such as pregnancy, migration stage and age. Humpback whales are a promising candidate for such validations due to widespread research and extensive individual-identification catalogues that exist for many populations (Fleming and Jackson, 2011). Fluke markings are unique and reasonably stable, such that photographs of the ventral side of the flukes can be matched over seasons and years to track individual whales across space and time (Katona *et al.*, 1979). Two of the longest running cetacean photo-ID catalogues are based on humpback whales from the Gulf of Maine (western North Atlantic Ocean) and Southeast Alaska, from which catalogues have been kept since the 1970s and 1960s, respectively (Gulf of Maine Humpback Whale Catalog, Center for Coastal Studies, Provincetown, MA; Gabriele *et al.*, 2017). Though it is rare to obtain baleen from an individual humpback whale with a known sighting history, some such cases do exist; thus, this species is a promising candidate for comparison of multi-year physiological information from baleen with past sighting histories.

In this first study of baleen hormones in humpback whales, we assess progesterone profiles in the baleen of four individuals with detailed sighting records: two adult females with known pregnancies, one adult female with a known history of no calves and one juvenile male for comparison. The major objective of this study was to determine whether pregnancy can be diagnosed via patterns of elevated progesterone in humpback whale baleen, with the period of baleen growth determined from SI analysis.

## Materials and methods

### Study animals

We studied the baleen recovered during necropsy of three female humpback whales (two from Southeast Alaska: SEAK 68, SEAK 1473; and one from the Gulf of Maine, ‘Spinnaker’, NAHWC #8587) and one male humpback whale from the Gulf of Maine (‘Lighthouse’, NAHWC #9464) (see Table 1 for baleen and body lengths). Southeast Alaska (SEAK) whales are referred to hereafter by their SEAK catalogue numbers (SEAK 68, SEAK 1473) but Gulf of Maine whales are referred to primarily by their names (Spinnaker, Lighthouse), for consistency with other reports on these individuals.

**Table 1:** Age, sex, sampled baleen length and body length in four humpback whales (*M. novaeangliae*); age (in years) was determined by sighting records or earplug analysis

ID	Age	Sex	Baleen length (cm)	Body length (m)
SEAK 1473	~13	Female	64	~12.5
SEAK 68	44.5	Female	66	13.87
Spinnaker	11	Female	66	10.79
Lighthouse	3	Male	45	10.97

SEAK 68 was an adult female from the North Pacific population that was first seen in 1975 and was subsequently sighted in Alaska 11 times and Hawai’i twice (Gabriele *et al.*, 2010). According to her sighting history, she was seen with a calf five times. She was found dead in July 2001 and upon examination she was determined to be pregnant (foetus length, 39.2 cm), with cause of death identified as ship strike (Gulland, 2001). Earplug analysis, which allows for ageing an individual, indicates she was ~44.5 years old (Gabriele *et al.*, 2010).

SEAK 1473, the other pregnant female, was also from the North Pacific population and was first spotted in Glacier Bay in 1997 and subsequently only ever photo-identified in that location. She was seen every year from 1997 until 2008, found dead in Glacier Bay in May 2010, and was found at necropsy to be pregnant. She was only seen with a calf once, in 2007. SEAK 1473 appeared to be a ‘small’ humpback whale in sightings during 1998–2000 and she strongly resembled a calf that was documented in Glacier Bay in 1996 but it was not possible to confirm this match, or her exact age. Due to advanced decomposition of the carcass, her cause of death is unknown. Because of the large size of her foetus (168 cm) and typical parturition of humpback whales during winter, it is thought that she died in October 2009 and remained on the beach over the winter until she was found in spring 2010.

Spinnaker and Lighthouse were both from the North Atlantic population and had sighting histories in the Gulf of Maine. Spinnaker was first catalogued there as a calf in 2004 and was observed in every subsequent year until her death at age 11 in 2015. She had an unusually elaborate history of human interactions, all well documented by regular sightings and entanglement response interventions. Although Spinnaker had surpassed the earliest known age (5 years old) of sexual maturity for humpback whales in this population (Clapham 1992; Robbins, 2007), she had not yet been observed with a calf. Lighthouse was a male that was 3 years old at death and therefore had most likely not reached male sexual maturity (>6 years old) (Chittleborough, 1955; Clapham, 1992). His cause of death is unknown (MAL-03-0303-Mn/NEAQ MH-03-602-Mn report reference). He is included in this study as an example of a young male who would be expected to have low progesterone as compared to mature females.

### Measurement of baleen plates and estimating date of growth or pregnancy

Baleen was removed from each whale by either cutting out a portion of the gum tissue to allow for extraction of the full plate or collected from the necropsy site after the plate fell out of the mouth. Baleen plates were cleaned of gum tissue if necessary; cleaning was done by separate necropsy teams for each whale. The plates were then stored in a heated building (SEAK 68 and 1473) or an indoor laboratory (Spinnaker and Lighthouse) until shipment to the laboratory in 2018, after which all specimens were sampled. Baleen plates were measured by permanently attaching tape (marked every 1 cm) along the posterior face of each plate, ~2 cm from the labial edge (see Hunt *et al.*, 2016a).

The proximal end of the base of the plate (newest baleen) that was embedded in the gum tissue was designated as the ‘zero cm’ point and was assigned an estimated growth date of the day before the whale was found dead (or, in SEAK 1473’s case, the date of death was considered 1 October of the prior fall, due to foetal length). Points along the baleen plate were then assigned an estimated date-of-growth based on the distance from the zero-cm point and the estimated BGR, determined via SI analysis. Both pregnant females had two pregnancies believed to have occurred during the growth period of their baleen plate, the more recent pregnancy confirmed by foetal presence at necropsy and also a prior pregnancy inferred from sightings with a calf in previous years. Pregnant periods for the whales were separated into first and second pregnancies, since foetus lengths were available for the second pregnancies from necropsy information. For the first pregnancies (older in time), conceptions and births were both expected during December–January when females begin to give birth in the North Pacific (Darling, 2001). Age of the foetus was estimated from foetal growth rates (Berta *et al.*, 2016), allowing month of conception to then be assigned to a given point on the plate using foetal age and BGR as determined via SI.

### SI analysis

SI analysis was performed on baleen of the two known-pregnant females (funding limitations prevented SI analysis on the other whales). For the two pregnant females, 1.0 ± 0.2 mg of baleen powder from each sampling location (i.e. every 2 cm) was weighed directly into tin capsules before SI analysis. Samples were analysed for relative abundance of SI of carbon and nitrogen [expressed as δ^13^C and δ^15^N, respectively, and given as permil values (‰.)] using a Thermo FlashSmart elemental analyser in line with a ThermoFinnigan DeltaPlus XP continuous-flow isotope ratio mass spectrometer (Thermo Scientific, Bremen, Germany). Measured δ^13^C and δ^15^N values obtained from hair sample analysis were scale calibrated based on contemporaneously analysed isotopic reference materials of accepted δ values relative to the appropriate reference scale acting as scale anchors. The isotopic reference materials used were supplied by the International Atomic Energy Agency (IAEA-N-1, δ^15^N = 0.4 ± 0.2‰; IAEA-CH-7, δ^13^C = −32.151 ± 0.050‰; IAEA-CH-3, δ^13^C = −24.724 ± 0.041‰) and the United States Geological Survey (USGS25, δ^13^C = −34.58 ± 0.06‰, δ^15^N = −0.94 ± 0.16‰; USGS40, δ^13^C = −26.389 ± 0.042‰, δ^15^N = −4.5 ± 0.1‰; USGS41, δ^13^C = +37.626 ± 0.049‰, δ^15^N = 47.6 ± 0.2‰;). Internal laboratory standards were included with all samples as quality controls; all error data are SD (purified methionine, Alfa Aesar, δ^13^C = −34.58 ± 0.06‰, δ^15^N = −0.94 ± 0.16‰; homogenized Chinook salmon muscle, NOAA Auke Bay Laboratories, δ^13^C = −19.27 ± 0.05‰, δ^15^N = 15.56 ± 0.13‰). Long-term records of internal standards yield an analytical precision (standard deviation) of 0.10‰ for δ^13^C and 0.15‰ for δ^15^N. Analysis of hormonal periodicity focused on nitrogen isotope ratios because nitrogen isotopes have been reported to have more pronounced and predictable annual variation in baleen whales (Best and Schell, 1996; Busquets-Vass *et al.*, 2017; Lysiak, 2008; Lysiak *et al.*, 2018; Matthews and Ferguson, 2015).

BGR for SEAK 68 and SEAK 1473 was estimated from δ^15^N values using a smoothing function (4 nearest neighbours) and calculating the distance between each peak. A peak was determined by using the localized maximum average δ^15^N levels; distance between was measured to the next peak, with a minimum distance to the next peak at 5 cm so that it was outside the moving average. Data from other whale species indicate that BGR is relatively constant for adults but is faster in juveniles (e.g. Schell, 2000). Therefore, BGR for the two other whales (Lighthouse, 3 years old; Spinnaker, 11 years old) was tentatively estimated based on age and on the empirically calculated BGRs of SEAK 68 (aged 44.5 years, average 17 cm/yr) and SEAK 1473 (aged ~13 years, average 20 cm/yr). Specifically, because Lighthouse and Spinnaker did not have SI information and were much younger than SEAK 68 (by ~42 and ~34 years, respectively), they were assigned a BGR of 20 cm per year.

### Homogenization of baleen and extraction of hormones

Extracts were prepared using a hand-held electric rotary grinder (Dremel Model 395 Type 5) to abrade a short (<1.5 cm) transverse groove across the posterior face of the plate, starting at ‘zero cm’, with baleen powder collected on a piece of weigh paper. Successive samples were collected every 2 cm along the complete length of the plates. Based on typical BGR in related baleen whales (between 16 and 24 cm/yr, depending on species and age class), we expected that this sample spacing should result in 8–12 samples per year (Hobson *et al.*, 2004; Rogers *et al.*, unpublished data). Total number of baleen subsamples was 30 for SEAK 68, 31 for SEAK 1473, 33 for Spinnaker and 23 for Lighthouse. Due to damage at the root (newest baleen, embedded in gum tissue) of some of the plates and additional use of extracts for performing reruns, not all plates have results every 2 cm; for example, SEAK 1473 had extensive damage at the gumline preventing hormone analysis until 4 cm into the plate. If necessary, powder from either side of the desired location was sampled instead (e.g. if the 22 cm sampling point could not be assayed, 21 or 23 cm was measured). Following previous methodology used successfully in other species (Hunt *et al.*, 2016a; Hunt *et al.*, 2017a; Hunt *et al.*, 2017b, Hunt *et al.*, 2018), hormones were extracted with 4.00 ml 100% methanol added to 75.0 mg of homogenized baleen powder, vortexed (2 hours), centrifuged for 5 minutes and then 3.00 ml of resultant supernatant was re-extracted and dried down. Extracts were reconstituted in 0.50 ml assay buffer (‘progesterone assay buffer’ #X065, Arbor Assays, Ann Arbor, MI, USA), sonicated 5 minutes and then shaken for 5 minutes to aid resuspension, transferred to cryovials, cooled and then decanted to a new cryovial to remove any remaining particulates. This produced a ‘1:1’ or full-strength extract. All extracts were stored at −80°C and assayed for progesterone within 1 year.

### Hormone assays

Complete methodology can be found in Hunt *et al.* (2016a). All samples were assayed with a commercial progesterone enzyme immunoassay kit (catalogue #K025-H1; Arbor Assays). This assay was selected based on previous successful assay validations (parallelism and accuracy) using powder from one of our study individuals, Spinnaker, as well as successful validation in seven other species of baleen whales (Hunt *et al.*, 2017b). Based on percent-bound in pilot data, all samples were diluted 4-fold in assay buffer for the initial assay so as to fall close to 50% on the standard curve, the area of greatest assay precision.

The manufacturer’s protocol was followed with one exception, adding an additional low-dose standard via extension of standard serial dilution to produce an eighth standard, with the final standard curve spanning 25–3200 pg/ml. The manufacturer’s reported sensitivity limit is 47.9 pg/ml, intra-assay precision is <5.1% and inter-assay precision is <7.0%; for humpback whale baleen, intra-assay precision was 3.9% and inter-assay precision was 5.7% (Hunt *et al.*, 2017b). For further details, including antibody cross-reactivities, see Hunt *et al.* (2014).

Samples were randomly assigned to wells and assayed in duplicate at 1:4 dilution (established from pilot data), and non-specific binding and blank wells were assayed in quadruplicate. Any samples with extremely high progesterone (percent bound <5%, *n* = 30) were then diluted to 1:16 or 1:64 and re-assayed to bring them closer to 50% bound on the standard curve. Samples with >10% coefficient of variation between the two duplicates (*n* = 24) or that had anomalous results compared with neighbouring samples were re-assayed (*n* = 11). Final results were converted to nanograms of immunoreactive progesterone per gram of baleen powder using GraphPad Prism 8.0.1., and results were graphed using R 4.0.2 (R Core Team 2020).

## Statistics and data analysis

To understand how pregnancy status affected progesterone levels both between the two reproductive females (SEAK 68 and SEAK 1473) and within the baleen growth period, we used mixed linear effects (nlme package; Bates *et al.*, 2014) to model the dependent variable (total progesterone concentration) as a function of pregnancy status (categorical with two levels: pregnant or non-pregnant). Samples were assigned pregnancy status based on back-calculations along the baleen in accordance with sighting histories showing the females with a calf. As samples were obtained from a single baleen plate for each individual, we included individual (ID: SEAK 68 or SEAK 1473) and the distance from baseline (cm) from each sample as random factors. The models were constructed as follows:

Progesterone concentration ~ Pregnancy status +, random = list(~1|ID, ~1|cm).

To obtain the significance of the fixed effect, we used the ‘summary(model)’ function in R and considered the factor to be significant when *t*-values were >2 and <−2.0 and the *P*-value was <0.05 (Luke, 2017). Z-scores were calculated using the ‘dplyr’ package (Wickham *et al.*, 2018) where a z-score for each sample along the baleen plate was calculated as ((absolute progesterone concentration − mean of progesterone for full plate)/standard deviation of full plate). A z-score of one indicates that the concentration of progesterone at that sampling point was one standard deviation higher than the average progesterone of the full baleen plate.

## Results

### SI results and BGR

For SEAK 68 (age 44.5), three full annual cycles were apparent from SI data with partial additional cycles; the baleen plate spanned an estimated ~3.5 years of baleen growth with four total SI peaks. SEAK 68’s average annual BGR was 17 cm/yr (20 cm for first full year on the baleen plate, 16 cm during year two, 15 cm during year three). SEAK 1473 had similar length baleen as SEAK 68 (also ~3.5 years of growth total), but because SEAK 1473 died when δ^15^N was low, only two full years of growth could be analysed via SI data. The average annual BGR for SEAK 1473 was 20 cm/yr (18 cm in first full year on the baleen plate and 22 cm in year two). These growth rates are similar to averages determined for North Pacific humpback whales in a separate study (Rogers *et al.*, unpublished data).

### Baleen progesterone

SEAK 1473’s foetus was estimated to be 8 months old (168 cm) and SEAK 68’s foetus was estimated to be 4 months old (39.2 cm) based on foetal growth rates in Berta *et al.* (2016), implying a likely conception month of February for SEAK 1473 and March for SEAK 68.

**Figure 1:**
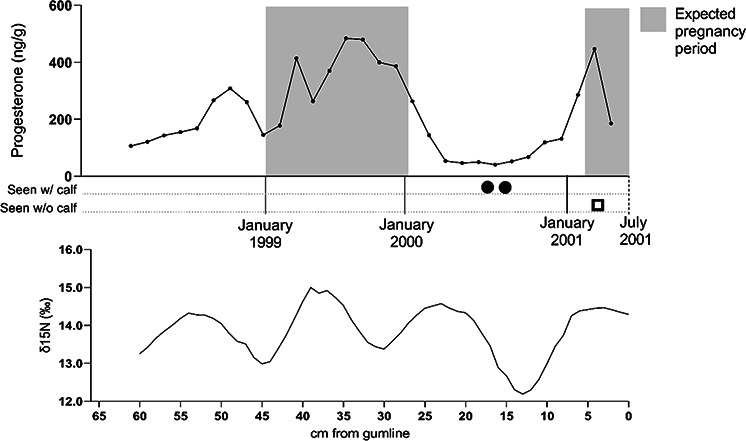
Baleen progesterone concentrations (top) and δ^15^N (bottom) from 1998 to 2001 for female humpback (*Megaptera novaeangliae*) SEAK 68. Shaded grey boxes show estimated pregnancy period based on 12-month gestation with conception and calving occurring in January (Clapham, 2000). The x-axis on the top graph depicts estimated dates of growth of each point along the baleen plate, derived from the distance from the proximal-most point on the plate (at base, newest baleen) and the estimated BGR (from SI data). Monthly sightings records are shown below the top graph with number of shapes denoting number of sightings: female sighted with neonatal calf, shaded circle; female sighted without a calf, open box (sighting data from Jan Straley and Glacier Bay National Park; for full sighting history, see Gabriele *et al.*, 2010). SEAK 68 was pregnant at death, determined to have a 39.2-cm foetus at post-mortem examination and was 44.5 years old based on earplug analysis.

For progesterone analysis, a mixed model with individual and pregnancy status factors was significant (LMM: df = 83, *t* = 15.72, *P* < 0.001), indicating that progesterone concentrations were greater during pregnancy than non-pregnancy. SEAK 68’s full baleen plate progesterone concentrations ranged from 40.78 to 484.14 ng/g with z-scores ranging from −1.27 to 1.90 (mean = 218.09 ng/g, median = 173.15 ng/g; Fig. 1; Table 2). At ~3 years before death, progesterone was relatively low but there was a small rise in progesterone concentrations from 50 to 46 cm; these values occurred during non-pregnancy (according to back calculations using BGR) and most likely occurred in early fall 1998 based on SI analysis. During the period between 42 and 32 cm, the area likely corresponding to the first of her two pregnancies (since she was seen with a calf in summer 2000 and typical conceptions occur in winter), progesterone started increasing (approximately January 1999) and then peaked approximately three-quarters of the way through the calculated gestation before decreasing shortly after the birth in December 1999 (Fig. 1). Concentrations stayed low and stable for ~1 year until climbing again in winter (late 2000, just prior to conception of her second pregnancy), peaking at 4 cm along the baleen plate when the expected period of conception occurred, followed by a decline. Overall, progesterone concentrations rose during pregnancy and, while variable, stayed relatively elevated compared to non-pregnant periods for the expected 12-month duration of gestation.

**Table 2:** Progesterone (ng progesterone per gram of baleen powder) averages and standard deviations with number of samples (*n*) for four humpback whales (*M. novaeangliae*)

ID	Pregnant	Not pregnant	Full plate
SEAK 1473	600.27 ± 159.88 (10)	102.44 ± 134.74 (21)	263.0 ± 275.20 (31)
SEAK 68	352.09 ± 111.52 (11)	140.51 ± 85.02 (19)	218.09 ± 139.77 (30)
Spinnaker	NA	NA	21.45 ± 14.97 (33)
Lighthouse	NA	NA	24.97 ± 14.99 (23)

Both SEAK 68 (age 44.5) and 1473 (age ~13) had two pregnancies and their progesterone levels are summarized during their estimated pregnant and non-pregnant periods.

Spinnaker (age 11) was never pregnant and Lighthouse (age 3) was male.

**Figure 2:**
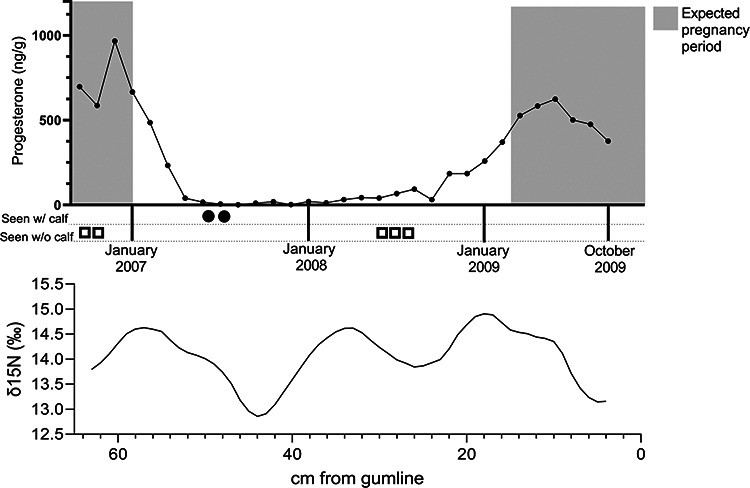
Baleen progesterone concentrations (top) and and δ^15^N (bottom) from 2006 to 2009 for female humpback (*Megaptera novaeangliae*) SEAK 1473. Shaded grey boxes show estimated pregnancy period based on 12-month gestation with conception and calving occurring in January (Clapham, 2000). The x-axis on the top graph depicts estimated dates of growth of each point along the baleen plate, derived from the distance from the proximal-most point on the plate (at base, newest baleen) and the estimated BGR (from SI data). Monthly sightings records are shown below the top graph with number of shapes denoting number of sightings: female sighted with neonatal calf, shaded circle; female sighted without a calf, open box. SEAK 1473 was pregnant at death, determined to have a 168-cm foetus at post-mortem examination and was ~13 years old based on sighting data.

SEAK 1473’s baleen progesterone concentrations ranged from 1.09 to 966.46 ng/g with z-scores ranging from −0.95 to 2.56 (mean = 263.03 ng/g, median = 184.85 ng/g; Fig. 2; Figure S1; Table 2). At the oldest part of the baleen, progesterone content was mid-range and then increased until a peak at 60 cm; this occurred during the period of expected conception of her first pregnancy. Progesterone then decreased and remained relatively stable for ~2 years before rising again and reaching its second peak at 10 cm during the period of estimated conception of her second pregnancy. There was a gradual decline in progesterone at the end of the second pregnancy after expected conception.

**Figure 3:**
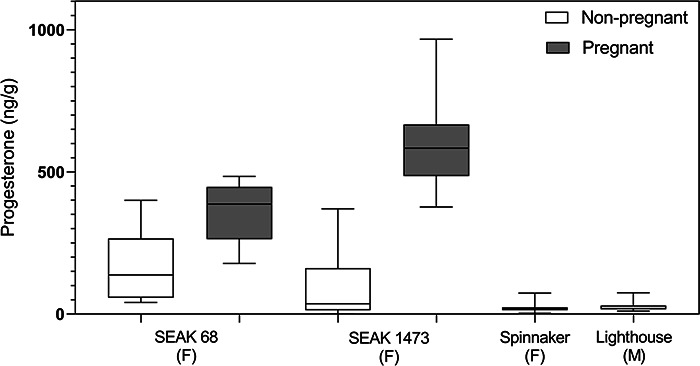
Concentration of baleen progesterone per gram of powdered humpback whale (*Megaptera novaeangliae*) baleen during non-pregnancy (light) and pregnancy (dark). X-axis is individual whale with sex, male (M) or female (F). SEAK 1473 was ~13 years old; SEAK 68, 44.5 years old; Spinnaker, 11 years old; Lighthouse, 3 years old. Each box ranges from the first quartile to the third quartile. The median is indicated by a line across each box, and the whiskers extend from the first and third quartile to the most extreme data points.

Progesterone concentrations during periods when these two whales had confirmed pregnancies were significantly higher than during non-pregnant periods (Fig. 3). Mean progesterone concentrations during pregnancy for SEAK 1473 (~13 years old) were 524.96 ng/g and for SEAK 68 (44.5 years old) were 361.88 ng/g. The peak progesterone level during pregnancy was twice as high for the younger whale than the older whale (966.46 ng/g vs. 484.14 ng/g).The non-pregnant levels between the two healthy females also differed, with the older female having higher mean non-pregnant progesterone concentrations (132.44 ± 79.64 ng/g) than the younger female (104.44 ± 140.86 ng/g). The average z-scores for each whale differed depending on pregnancy status. For SEAK 68, average z-score was 0.92 ± 0.77 during pregnancy and −0.61 ± 0.57 for non-pregnancy. For SEAK 1473, average z-score was 1.21 ± 0.59 during pregnancy and −0.58 ± 0.51 during non-pregnancy (Figure S1).

The other two individuals, nulliparous female Spinnaker and juvenile male Lighthouse, had consistently low progesterone across the length of their baleen plates. Spinnaker had progesterone values that ranged from 2.61 to 73.87 ng/g (mean = 21.45 ± 14.97 ng/g, z-scores = −1.3 to 3.5) (Fig. 3; Figure S1; Table 2). Lighthouse had similar progesterone values from 10.50 to 74.88 ng/g (mean = 24.97 ± 14.99 ng/g, z-scores = −0.16 to 3.3) (Fig. 3; Figure S1; Table 2). Neither whale had elevated progesterone that spanned more than one sample point. Lighthouse had one ‘spike’ (brief elevation at a single sampling point, but not in neighbouring sampling points) at 39 cm and Spinnaker had two single point spikes, at the beginning and end of the plate (1 cm and 63 cm). Maximum progesterone of Lighthouse and Spinnaker (~75 ng/g for both) was ~6.5 and 13 times lower than the maxima observed in SEAK 68 (484.14 ng/g) and SEAK 1473 (966.46 ng/g), respectively.

## Discussion

Generally, pregnancy was associated with baleen progesterone concentrations of >100 ng/g and confirmed pregnancies tended to exceed this threshold throughout the duration of the pregnancy. In contrast, baleen from a nulliparous female and a juvenile male did not have progesterone concentrations >75 ng/g and never showed prolonged (more than one sampling point) elevations, whereas the average concentrations for the two reproductive females during pregnancy were much higher at 352.09 ng/g and 600.27 ng/g (see Table 2). Although this study only included two pregnant whales, the results from this study and previous studies on NARWs and bowheads show increases in baleen progesterone during pregnancy (Hunt *et al.*, 2016a; Kellar *et al.*, 2013) and highlight that baleen could be a useful tissue type to examine pregnancy intervals over multiple years. Further analysis should include additional confirmed pregnant females across multiple ages to determine progesterone patterns during pregnancy as whales age and how they vary by individual.

We suggest that in humpback whale baleen, progesterone concentrations that are at least 100% above baseline (full plate average) and that last for more than 10 months of baleen growth likely indicate a full-term pregnancy. Z-scores were reported so future baleen analysis can be compared to determine if progesterone concentrations suggest pregnancy. The methods used to quantify hormones in this study can determine inter-calving intervals and total numbers of pregnancies that occurred during the baleen growth period in humpback whales. Though only four individuals could be assessed, each plate was intensively subsampled and our data indicate that humpback whale baleen not only accurately reflects recent pregnancy, but that it captures a sufficient temporal interval to capture a complete inter-calving interval (most recent pregnancy, non-pregnant interval and at least part of the prior pregnancy).

The ‘pregnancy signature’ of high progesterone in humpback whale baleen is remarkably pronounced and has been documented in two other baleen whales, NARW and bowhead whales, both of which show progesterone levels up to two orders of magnitude higher in baleen grown during pregnancies, as confirmed by sighting and necropsy data (Hunt *et al.*, 2014; Hunt *et al.*, 2016a). NARWs and bowhead whales are closely related (family Balaenidae) and have unusually lengthy and slow-growing baleen that is long enough to capture multiple inter-calving intervals and pregnancies (up to 10 years or more of growth; Hobson *et al.*, 2004; Hunt *et al.*, 2014). Prior to this work, it was unknown if the shorter baleen in humpback whales could capture multiple reproductive states or if the progesterone levels in baleen would be similar between multiple species, but we have confirmed that the pregnancy signature is remarkably similar between bowheads, NARWs, and humpback whales. However, the concentrations of progesterone during pregnancy in the baleen of humpback whales are lower than the levels seen in NARWs and bowheads (>1000 ng/g; Hunt *et al.*, 2014, 2016a), especially in the older female (SEAK 68). The pattern of decreasing progesterone at the end of life during pregnancy in both whales in this study were also seen in NARWs (Hunt *et al.*, 2016a) and might reflect changes in extraction efficiency in baleen along the plate or decreases in the binding affinity of the metabolites from newer baleen to the assay antibody. We recommend a follow-up study to assess if this pattern occurs consistently in other species in which an individual whale has at least two pregnancies documented in the same baleen plate.

Each individual’s baleen in this study generated a multi-year dataset; that is, repeated sampling spanning 3–4 years. The resulting SI cycles and progesterone profiles showed clear correspondence to past sightings records, with prolonged elevations of progesterone matching known pregnancies and SI matching expected seasonal feeding patterns. The SI results agree with previous findings on general BGRs, but individual assessment is advised to determine exact dating as there is year-to-year variation in baleen growth. SI-derived estimates of BGR had not previously been published for Northern Hemisphere humpback whales specifically, making retrospective extrapolation of events difficult; Eisenmann *et al.* (2016) found that the BGR for Southern Hemisphere humpback whales ranged from 12 to 20 cm. BGRs for the two whales in this study averaged 20 cm/year (~13 years old) and 17 cm/year (44.5 year old), with the younger whale having faster baleen growth, as is typical in baleen whales (Eisenmann *et al.*, 2016; Rogers *et al.*, unpublished data). It is possible that BGR may vary slightly across a year, altering the apparent timeline that is extrapolated from the sighting history, but those variations should not affect the overall pattern of progesterone required for pregnancy diagnosis. At present, SI cycles are still necessary to establish the duration of inter-calving intervals; however, as further studies refine our estimates of BGR in humpback whales from different populations of various sexes and ages, combined with studies of age to body length ratios, it will likely become possible to assign baleen plates an estimated timeline solely based on population, whale sex and body length (Stevick, 1999).

During pregnancy, overall progesterone concentrations were lower in the older female (44.5 years old) than in the younger female (~13 years old), which may be due to individual differences in hormone secretion or deposition into baleen. It may, however, be related to the age difference between the two whales. Humpback whales have been documented to live up to 96 years (Chittleborough, 1959). Because both females had prior pregnancies that resulted in live calves that survived into the summer season, evidently the low progesterone content in the older female during pregnancy did not affect reproductive success. There is evidence for possible reproductive senescence in some baleen whales, such as declining testosterone in baleen of older male bowhead whales (Hunt, pers. obs.) that could account for the older whale’s lower progesterone levels, but there is currently no known evidence of reproductive senescence in female humpback whales. In addition to the differences between progesterone concentrations between the two whales, there was also a decrease in progesterone during the second pregnancies in both whales that occurred shortly before death. Since both of these decreases took place in newly grown baleen, it is unlikely that this is the result of degradation of the plate but could be due to extraction efficiency differences in baleen that is close to the gumline.

This study also provides the first longitudinal data on the pregnancy status of a female humpback whale (Spinnaker) whose sub-adult years were marked by multiple entanglements in fishing gear. When comparing the difference in progesterone concentrations between the two presumably healthy mature females (SEAK 68 and SEAK 1473) with the repeatedly entangled nulliparous female (Spinnaker), the latter had lower average progesterone throughout the entirety of the baleen growth period (79% and 84% lower than SEAK 68 and SEAK 1473, respectively). A complicating factor is that the reproductive status of Spinnaker has some uncertainty. She was never observed with a calf, despite having exceeded the minimum age (5–9 years) at which sexual maturity can occur in this species (Clapham, 1992). This lack of a calving history suggests, but cannot absolutely confirm, that she never became pregnant. Lack of a calf sighting history is not necessarily unusual relative to other female members of her birth cohort, although sample sizes are limited (Center for Coastal Studies, unpublished data). However, it is also possible that her entanglement history delayed attainment to sexual maturity or impacted her ability to maintain a pregnancy; further study of baleen glucocorticoids could illuminate the possible effect of entanglement stress on reproduction. She was also entangled frequently enough to impair locomotion, foraging and migration, all of which are vital for reproduction, as fat stores are needed to successfully rear a calf and migrate to the breeding grounds. Indeed, she was described as being small for her age in sighting records (Center for Coastal Studies, unpublished data). Inter-calving intervals have been shown to lengthen dramatically (five or more years) in injured or nutritionally stressed NARWs when additional ‘resting’ (non-pregnant or lactating) years are presumably needed to replenish fat stores (Angell, 2006; Hamilton and Cooper, 2010; Hlista *et al.*, 2009).

According to whaling data, humpback whales typically have a 2-year calving interval (Chittleborough, 1958; Matthews, 1937), although whaling data often overestimated calving intervals because whalers were not permitted to hunt lactating females (Lockyer, 1984). However, using a 30-year time series, Gabriele *et al.* (2017) found a 3.1–3.3-year interval for the North Pacific population that was somewhat longer than previously published values for this species (Baker *et al.*, 1987). For the Gulf of Maine population, average observed calving intervals have generally ranged from 2 to 3 years (Clapham and Mayo, 1987; Clapham and Mayo, 1990; Clapham *et al.*, 2003; Barlow and Clapham, 1997). There are cases of calves born 1 year apart as well as intervals of >5 years (Baker *et al.*, 1987; Clapham and Mayo, 1987; Clapham and Mayo 1990, Barlow and Clapham, 1997; Straley *et al.*, 1994; Weinrich *et al.*, 1993). SEAK 68 had a 2-year calving interval prior to death and SEAK 1473 had a 3-year interval, both of which fall into previously published records of 2–3 year intervals. As more baleen is analysed, the ranges in inter-calf intervals in individuals can be more precisely determined and will provide necessary data to better understand and predict population growth or decline.

The progesterone patterns seen here in the baleen of pregnant humpback whales agrees with findings from other sample types. Blubber analysis in humpback whales from the Gulf of Maine showed pregnant females have higher progesterone by two orders of magnitude (Pallin *et al.*, 2018) and bowhead whale blubber analysis found progesterone to be the highest in pregnant individuals, followed by non-pregnant adult females, then subadults (Kellar *et al.*, 2013). Blue whale (*Balaenoptera musculus*) and humpback whale faecal analyses showed higher levels of progestins during pregnancy than during non-pregnancy (Valenzuela-Molina *et al.*, 2018; Hunt *et al.*, 2019). Thus, a growing body of data indicates that pregnant baleen whales consistently show high progesterone (or related metabolites) in a variety of sample types, enabling pregnancy diagnoses through a variety of methods.

Since progesterone is important for the establishment and maintenance of pregnancy, increases during pregnancy are expected (Arck *et al.*, 2007). This pattern can be seen in many artiodactyls, the closest terrestrial relatives of whales, although peak levels and the general profile across gestation differ with species. The general pattern of progesterone throughout pregnancy in many mammalian species shows slowly increasing progesterone throughout pregnancy before reaching a peak that is substantially higher than non-pregnancy levels (Schwarzenberger and Brown, 2013); where this peak occurs is dependent on species. For example, in Asian elephants (*E. maximus*), progesterone peaks approximately halfway through the pregnancy and then steadily decreases until parturition (Kajaysri and Nokkaew, 2014). In pygmy hippos (*Choeropsis liberiensis*), progestogen metabolites slowly increase throughout pregnancy and do not peak until parturition (Flacke *et al.*, 2017). Further data will be necessary to explore these patterns in baleen whales (for example, comparisons of early pregnancy to late pregnancy). Other steroid hormones, such as testosterone, could also be useful indicators of pregnancy or impending birth (Dalle Luche *et al.*, 2020).

In the humpback whales in this study, progesterone started increasing during the winter season when mating behaviours occur and decreasing during the following winter season when calving occurs, supporting the hypothesis that elevated baleen progesterone coincides with gestation and is not due to other physiological processes such as fasting, growth or stress. It is currently unknown whether baleen whales are spontaneous or induced ovulators or how progesterone patterns change during ovulation. Progesterone might increase during periods of estrous in the winter but would not be sustained for many months without conception. Pseudopregnancy might also cause elevated progesterone in the absence of a foetus but this phenomenon has not been confirmed for baleen whales. Pseudopregnancies tend to be both shorter in duration and lower in overall progesterone concentration than true pregnancies (Renouf *et al.*, 1994; Robeck *et al.*, 2001). Extended post-ovulatory phases can also result in prolonged elevation of progesterone, though with shorter duration than a pregnancy (Brown, 2018). SEAK 68 had a short rise in progesterone that peaked at intermediate levels (133% above baseline) and lasted for ~3 months before decreasing. Afterwards, progesterone slowly increased once more, lasted for 12 months and peaked at 266% above baseline; this whale was sighted with a calf the following summer, confirming that the 12-month elevation in progesterone was due to true pregnancy. Based on the estimated 10–12 month gestation length of humpback whales (Chittleborough, 1955; Chittleborough, 1958), SEAK 68’s abbreviated rise in progesterone occurred before the documented pregnancy. This short rise may have been due to a luteal phase, estrous, pseudopregnancy or an early failed pregnancy. If the abbreviated peak was a failed pregnancy, this would indicate that humpbacks are capable of two ovulatory cycles within a single breeding season, as suggested by Chittleborough (1958).

Using progesterone profiles, along with other steroid hormones, historic baleen could be used to compare calving rates between historic populations (e.g. 1800s) and extant populations, investigating how anthropogenic activities and other variables may have altered reproductive rates in whale populations over time. Baleen is relatively easy to collect, it is easily dried and stored, and hormones in the baleen do not appear to degrade at room temperature, making it an ideal tissue type for hormone analysis. Hormones in bowhead whale baleen appear stable after up to 20 years of growth in the mouth (i.e. with continuous exposure to seawater) and 8 years of storage (Hunt *et al.*, 2014) and steroid hormones did not degrade in North Atlantic right whale baleen after 17 years in storage (Hunt *et al.*, 2018). Therefore, we encourage stranding response teams to routinely collect baleen when possible, as the hormone and SI profiles can be informative about the years prior to the death of the whale even if little information is known about the stranded individual. Other case studies involving baleen analysis with known sighting records are needed to assist in validating reproductive hormone patterns, allowing for future analyses of baleen with unknown histories. SI studies are needed to refine baleen growth estimates for the different humpback whale populations and age classes. Finally, the myriad steroid hormones involved in mammalian physiology should be tested in baleen, including metabolism, reproduction and stress hormones to elucidate individual and population health in this species. Using hormone profiles contained in baleen to determine calving intervals for humpback whales is a useful tool for determining multi-year physiological profiles for this long-lived, difficult-to-sample species.

## Funding

This work was supported by the Technology and Research Initiative Fund at Northern Arizona University.

## Permits

Baleen was collected under NOAA permit 932-1905-MA-009526. Laboratory analysis of baleen occurred under NOAA Marine Mammal Parts Authorization issued to K.E. Hunt.

## Trade Names Disclaimer

Reference to trade names does not imply endorsement by the National Marine Fisheries Service, NOAA.

## Disclaimer

The scientific results and conclusions, as well as any views or opinions expressed herein, are those of the author(s) and do not necessarily reflect those of NOAA or the Department of Commerce.

## Conflicts of interest

The authors declare that they have no conflicts of interest.

## Supplementary Material

S1_Z_scores_coab059
