# Supplementary figures and images for: Multi-year progesterone profiles during pregnancy in baleen of humpback whales (Megaptera novaeangliae)
Source: Conserv Physiol. 2021 Jul 28;9(1):coab059. doi: 10.1093/conphys/coab059 (PMC8567847; doi:10.1093/conphys/coab059)

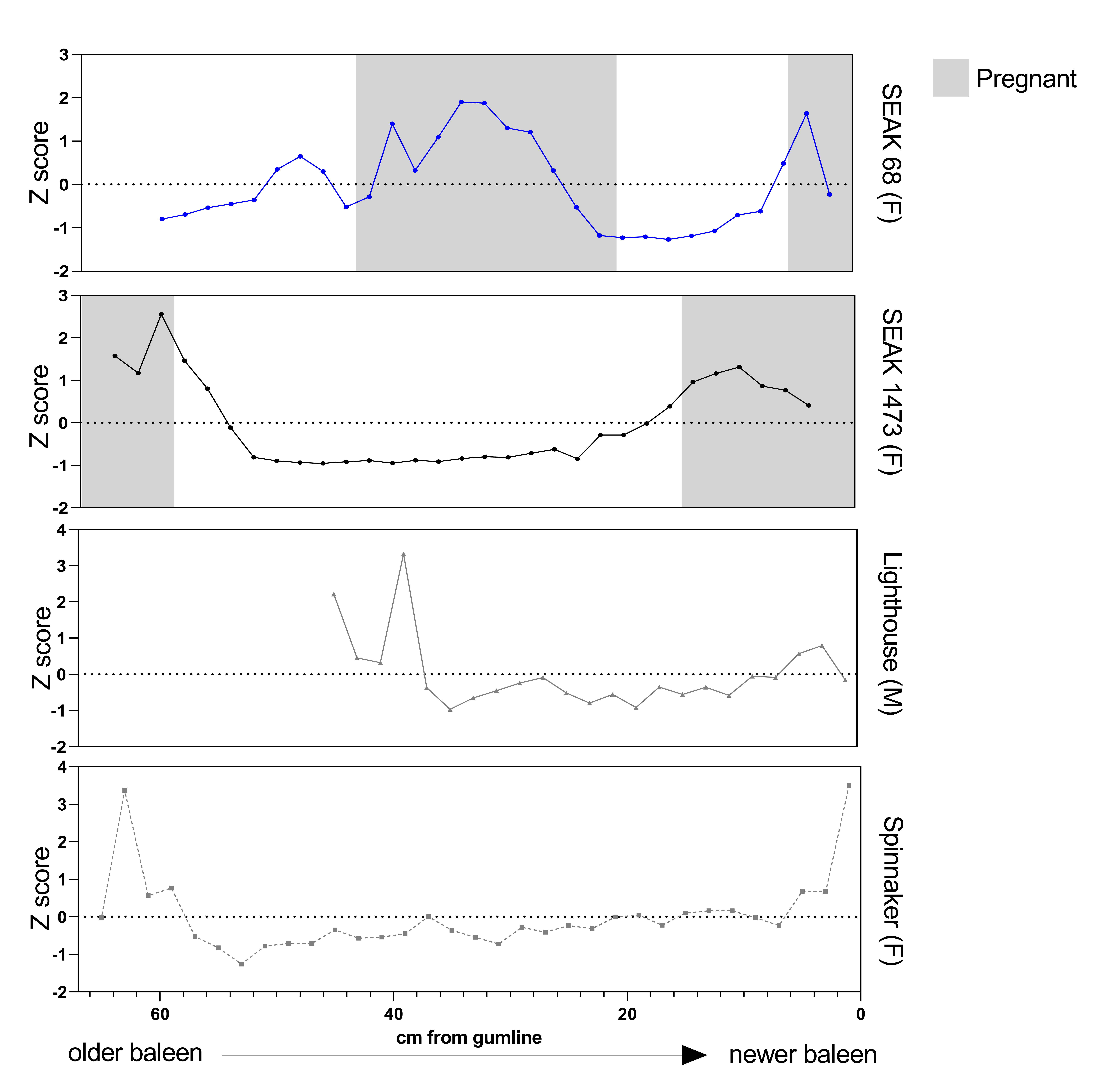

Supplement: S1_Z_scores_coab059 [file s1_z_scores_coab059.zip › S1_Z_scores_coab059.tif]
